# Sex-specific differences in the associations of metabolic syndrome or components with gallstone disease in Chinese euthyroid population

**DOI:** 10.1038/s41598-023-28088-z

**Published:** 2023-01-19

**Authors:** Li Jiang, Jinman Du, Jufang Wang, Jinhua Ding

**Affiliations:** 1 Department of General Medicine, Ningbo Medical Center Lihuili Hospital, Ningbo, 315000 China; 2 Health Examination Center, Ningbo Medical Center Lihuili Hospital, Ningbo, 315000 China; 3 Department of Breast and Thyroid Surgery, Ningbo Medical Center Lihuili Hospital, Ningbo, 315000 China

**Keywords:** Endocrinology, Gastroenterology

## Abstract

In euthyroid population, it is uncertain whether there is sex-specific difference in the associations of metabolic syndrome (MetS) or its components with gallstone disease (GSD); in general population, MetS increases the risk of GSD. This was a cross-sectional study to investigate the sex-specific difference in the prevalence of MetS according to GSD status and the associations of MetS or its components with GSD in Chinese euthyroid population. The total prevalence of GSD was 8.1% (6.5% in men and 11.0% in women, with a significant difference (*p* < 0.001)). The total presence of MetS was 10.7% (12.1% in men and 8.2% in women,with a significant difference (*p* = 0.001)). The age-adjusted odds ratio of MetS for GSD was 2.775 in men (*p* < 0.001), 2.543 in women (*p* = 0.007) and 2.503 in the oveall samples (*p* < 0.001). Univariate analysis revealed that fasting plasma glucose (FPG), high-density lipoprotein cholesterol (HDL-C) and thyroid-stimulating hormone (TSH) were associated with the prevalence of GSD. After adjustment for age, multivariate logistic regression analysis demonstrated that above three parameters were still significantly associated with the risk of GSD in general population; FPG and HDL-C but not TSH levels were significantly associated with the risk of GSD in men; and FPG and TSH levels but not HDL-C in women. Our study demonstrated that in euthyroid population, MetS appeared to be strongly associated with GSD regardless of sex, and FPG and TSH were two independent risk factors for GSD in men, while FPG and HDL-C in women.

## Introduction

Gallstone disease (GSD), as the most common disease of the biliary system, is often asymptomatic^1^. Nevertheless, it is associated with potential risks of cholecystitis, pancreatitis, cholangitis and even malignancy of gall bladder^2,3^. The prevalence of GSD varies among different populations and is higher in developed countries than in developing country such as China^4^, for example, 16.6% and 8.6% in non-Hispanic American women and men^5^, 18.4% and 9.5% in Italian women and men^6^ and 22.4% and 11.5% in British women and men^7^, respectively. Although the majority of studies conducted in Western population concluded that women were more likely to develop GSD than men^5–9^, studies among Asian individuals have failed to identify this difference in the prevalence of GSD by sex^10,11^.

Metabolic syndrome (MetS) is a group of metabolic diseases, including visceral obesity, hypertension, atherogenesis, dyslipidaemia and hyperglycaemia^12^. Numerous studies have shown a positive relationship between the presence of MetS and GSD^4,13–15^. Metabolic risk factors, or components of MetS, including hyperlipidaemia, high body mass index (BMI) and hyperglycaemia were closely related to GSD^16,17^ in a Western population. However, the associations between metabolic risk factors and GSD are still uncertain in Chinese population, only a few studies explored their potential association^18^. Whether there are sex-specific differences in the associations of MetS or components with GSD is also unknown.

Additionally, either hypothyroidism or hyperthyroidism was found to be associated with GSD^19,20^, and subclinical hypothyroidism was also associated with GSD^21–23^. However, in the population with normal thyroid function, the associations between MetS or components and GSD are unknown, and whether the level of thyroid stimulating hormone (TSH) is associated with the prevalence of GSD is also uncertain. Therefore, this study aimed to evaluate in Chinese euthyroid population: (i) whether MetS or its components are associated with the prevalence of GSD and (ii) whether there are sex-specific differences in the association of these factors with GSD.

## Results

### Demographic and clinical characteristics

A total of 2378 participants visiting the health examination centre of Ningbo Medical Centre Lihuili Hospital between June 2016 and December 2017 were included in this study. Among them, 65.2% (1551/2378) were male and 34.8% (827 /2378) were female. There were 192 participants with GSD, and the prevalence was 8.1% (192/2378). The prevalence of GSD was 6.5% (101/1551) and 11.0% (91/827) in men and women, respectively, and the difference was statistically significant (*p* < 0.001, OR = 0.563, 95% CI 0.419–0.758).

In female participants, there were significant differences in mean age (*p* < 0.001), SBP (*p* = 0.003), DBP (*p* = 0.029), TC (*p* = 0.008), LDL-C (*p* = 0.008), HDL-C (*p* < 0.001), TG (*p* < 0.001) and FPG (*p* = 0.029) between GSD and non-GSD groups (Table 1).In female participants, there were significant differences in mean age (*p* < 0.001), SBP (*p* = 0.003), TC (*p* = 0.007), LDL-C (*p* = 0.008), HDL-C (*p* < 0.001), TG (*p* < 0.001) and FPG(p=0.018) between GSD and non-GSD groups (Table 1).

**Table 1:** Participants’demographic and clinical characteristics in GSD and non-GSD group based on sex.

	GSD (Female) (n = 91)	non-GSD (Female) (n = 736)	GSD (Male) (n = 101)	non-GSD (Male) (n = 1450)	p*	p**	p***	p****
Age (years)	49.4 ± 8.3	43.4 ± 6.7	50.2 ± 8.2	44.0 ± 6.8	< 0.001	< 0.001	0.324	0.357
Height (cm)	160.5 ± 7.9	161.3 ± 7.5	168.2 ± 8.5	170.1 ± 8.7	0.423	0.058	< 0.001	< 0.001
Weight (Kg)	59.4 ± 11.0	56.4 ± 11.3	69.0 ± 12.3	70.1 ± 12.7	0.064	0.432	< 0.001	< 0.001
BMI (Kg/m^2^)	22.5 ± 3.3	22.1 ± 3.2	24.6 ± 3.3	23.9 ± 3.2	0.213	0.137	0.015	0.021
SBP (mm Hg)	119.3 ± 16.4	116.3 ± 15.3	126.2 ± 17.6	122.9 ± 17.4	0.015	0.003	< 0.001	< 0.001
DBP (mm Hg)	74.1 ± 11.2	72.2 ± 11.3	76.6 ± 11.5	74.9 ± 11.4	0.029	0.062	0.018	0.024
FPG (mmol/L)	5.0 ± 1.1	4.7 ± 1.0	5.3 ± 1.1	5.0 ± 1.0	0.021	0.018	0.024	0.020
TC (mmol/L)	4.8 ± 0.8	4.6 ± 0.9	5.1 ± 0.8	4.9 ± 0.9	0.008	0.007	0.003	0.003
TG (mmol/L)	1.9 ± 0.4	1.6 ± 0.3	1.6 ± 0.4	1.3 ± 0.3	< 0.001	< 0.001	< 0.001	< 0.001
HDL-C (mmol/L)	1.3 ± 0.3	1.5 ± 0.3	1.0 ± 0.3	1.2 ± 0.3	< 0.001	< 0.001	< 0.001	< 0.001
LDL-C (mmol/L)	3.1 ± 0.6	2.9 ± 0.8	3.1 ± 0.6	2.9 ± 0.8	0.008	0.008	0.653	0.712
fT3 (pmol/L)	5.1 ± 0.6	5.1 ± 0.7	5.1 ± 0.6	5.1 ± 0.7	0.781	0.834	0.682	0.715
fT4 (pmol/L)	15.9 ± 2.5	16.1 ± 2.4	16.2 ± 2.7	16.4 ± 2.4	0.087	0.092	0.060	0.056

Data were presented as mean ± SD. Groups were compared using t-test.

*BMI* Body mass index, *SBP* Systolic blood pressure, *DBP* Diastolic blood pressure, *FPG* Fasting plasma glucose, *TC* Total cholesterol, *HDL-C* High density lipoprotein cholesterol, *LDL-C* Low density lipoprotein cholesterol, *TG* Triglyceride, *fT3* Free triiodothyroxine, *fT4* Free tetraiodothyroxine.

*p* < 0.05 was considered as statistically significance. p* stood for the comparasion between GSD and non-GSD in female participants; p** stood for the comparasion between GSD and non-GSD in male participants; p*** stood for the comparasion of sex difference in GSD participants; p****stood for the comparasion of sex difference in non-GSD participants. *BMI: calculated as weight (in kilogram) divided by height (in meter) squared.

### Univariate analysis and multivariate logistic regression analysis

Univariate analysis revealed that in female population, high FPG (*p* < 0.001), low HDL-C (*p* = 0.027), and low TSH levels (*p* = 0.020) were associated with the prevalence of GSD; in male population, high FPG (*p* < 0.001), low HDL-C (*p* = 0.008), and low TSH levels (*p* = 0.026) were associated with the prevalence of GSD (Table 2).

**Table 2:** Comparison of metabolic risk factors and TSH between GSD and non-GSD group in female and male participants.

	Female participants			Male participants		
	GSD (*n* = 91)	Non-GSD (*n* = 736)	*p*	GSD (*n* = 101)	non-GSD (*n* = 1450)	*p*
Higher BMI						
Yes	33	239	0.468	37	456	0.279
No	58	497		64	994	
Higher BP						
Yes	26	191	0.592	30	330	0.114
No	65	545		71	1120	
Higher FPG						
Yes	25	81	< 0.001	23	104	< 0.001
No	66	655		78	1346	
Lower HDL-C						
Yes	21	105	0.027	24	197	0.008
No	70	631		77	1253	
Higher TG						
Yes	25	213	0.807	31	416	0.651
No	66	523		70	1034	
TSH level						
0.5–2.0	54	529	0.020	66	889	0.026
2.0–3.5	27	168		25	491	
3.5–5.0	10	39		10	70	

Data were presented as mean ± SD. Groups were compared using chi-square test. *p* < 0.05 was considered as statistically significance.

*BMI: calculated as weight (in kilogram) divided by height (in meter). squared.

After adjustment for age, multivariate logistic regression analysis demonstrated that above three parameters were still significantly associated with the risk of GSD in all participants; HDL-C and FPG, but not serum TSH levels were significantly associated with the risk of GSD in men; and serum TSH levels and FPG, but not HDL-C were significantly associated with the risk of GSD in women (Table 3).

**Table 3:** Age-adjusted multivariate logistic regression analysis for predicting GSD.

Variable	Adjusted OR	95% Confidence interval		
		Lower	Upper	*p*
Male				
TSH level	0.601	0.247	1.460	0.261
HDL-C	2.234	1.518	3.590	< 0.001
FPG	0.520	0.309	0.876	0.014
Female				
TSH level	0.329	0.145	0.744	0.008
HDL-C	2.130	0.871	5.208	0.098
FPG	0.086	0.047	0.157	< 0.001
Total				
TSH level	0.442	0.271	0.722	0.001
HDL-C	1.769	1.277	2.452	0.001
FPG	0.311	0.233	0.415	< 0.001

*OR* Odds ratio, *TSH* Thyroid-stimulating hormone, *HDL-C* High density lipoprotein cholesterol, *FPG* Fasting plasma glucose, *GSD* Gallbladder stone disease.

### Prevalence of MetS and the association with GSD

Among 2378 participants, there were 255 MetS patients, and the prevalence was 10.7% (255/2378). Among 255 patients with MetS, there were 187 and 68 men and women, respectively; the prevalence of MetS were 12.1% (187/1551) and 8.2% (68/857), respectively; and the difference was significant (*p* = 0.001, OR = 1.591, 95% CI 1.189–2.128). In age-adjusted logistic regression analyses, the prevalence of MetS was significantly associated with the risk of GSD irrespective of sex (Table 4). The age-adjusted OR of MetS for GSD was 2.775 (95% CI 1.726–4.464) in men, *p* < 0.001, 2.543 (95% CI 1.368–4.729) in women, *p* = 0.007, and 2.503 (95% CI 1.723–3.633) in the total samples, *p* < 0.001.

**Table 4:** Age-adjusted association of MetS with GSD by sex.

Gender status	GSD	Non-GSD	Adjusted OR (95%CI)	*p*
Male				
MetS	26	161	2.775 (1.726–4.464)	< 0.001
Non-MetS	75	1289		
Female				
MetS	15	53	2.543 (1.368–4.729)	0.007
Non-MetS	76	683		
Total				
MetS	41	214	2.503 (1.723–3.633)	< 0.001
Non-MetS	151	1972		

*GSD* Gallstone disease, *MetS* Metabolic syndrome, *OR* Odds ratio, *CI* Confidence interval.

**Figure 1:**
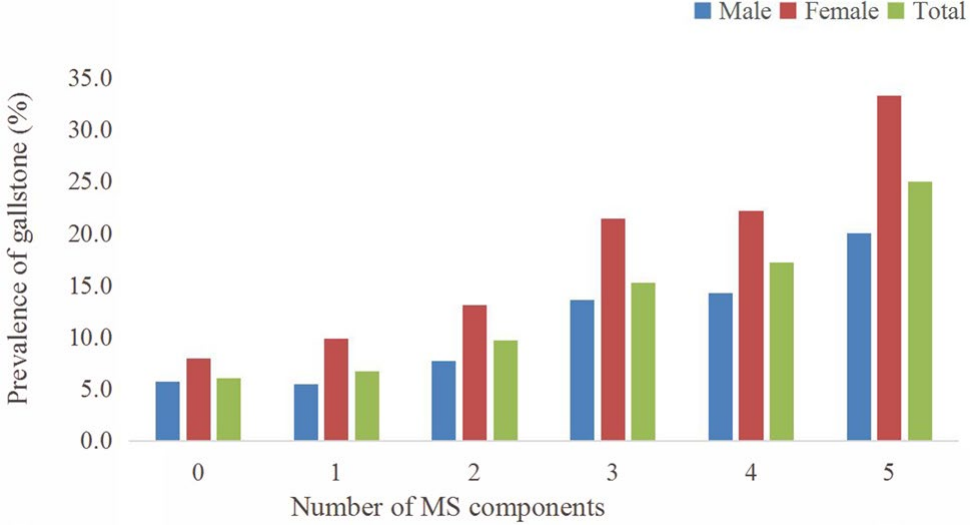
The association between the prevalence of GSD and the number of MetS components.

We also analysed the association between the prevalence of GSD and the number of MetS components. Figure 1 demonstrates these relationships. We found that the greater the number of components of MetS, the higher prevalence of GSD in men and in women, and the trend was significant (*p* < 0.001). In male participants, the presence of 5 components of MetS increased the risk of GSD by 3.5 times (20.0%/5.7%) when compared to those without any MetS component, and the prevalence of GSD in women who had 5 components of MetS was 4.2 times (33.3%/8.0%) that of those without MetS components. The trend in overall participants was also significant (*p* < 0.001).

## Discussion

The prevalence of GSD in our study was 8.1%, which is in line with previous studies in Asian populations, with rates ranging from 3 to 11%^3,24,25^. However, it was much lower than that in the Western population. The prevalence of MetS may contribute to explaining this difference. Numerous studies consistently reported a higher prevalence of MetS in Western populations than in Asian populations. Since the higher presence of MetS would inevitably result in a higher prevalence of GSD, the higher prevalence of GSD in the Western populations could be expected. For example, in an observational study conducted in Italy, the author reported a prevalence of 16.6% because all the subjects included had at least one major cardiovascular risk factor; thus, the prevalence of MetS reached as high as 51.2%.

In the current study, we found some sex-specific differences. First, the prevalence of GSD was different. The prevalence was much lower in men than in women, 6.5% vs. 11.1%, respectively (*p* < 0.001, OR = 0.563, 95% CI 0.419–0.758), which was consistent with most previous studies^8,26^. It is generally believed that sex hormones are related to cholesterol metabolism, indicating that sex may be related to cholesterol stones^13^. However, the results are always controversial. Some other studies indicated that sex was not related to GSD^2,20,21^, and even in a study conducted in mainland China, they found the prevalence in men was higher than in women (13.1% vs. 10.2%). Second, the prevalence of MetS was also different. In our study, the prevalence of MetS in men was higher than that in women, 12.1% versus 8.2%, respectively (*p* = 0.001, OR = 1.591, 95% CI 1.189–2.128), which was consistent with that in Caucasians^27^, American adolescents^28^, Far East Asian and Japanese populations, and Macau^29^. However, in other studies, the prevalence of MetS in women was higher than that in men^30–32^; for example, in Mexico, the prevalence in women was 55.6%, whereas in men, it was 38.2%^33^. Similar results were observed in Iran^34^. Third, although MetS was strongly associated with GSD regardless of sex, the risk factors for GSD in men and women were different. In our study, after age adjustment, low HDL-C and high FPG were confirmed to be independent risk factors for GSD in men while high TSH level and high FPG were in women.

In the total sample of our study, lower HDL-C and higher FPG were associated with gallstone formation. Previous studies showed that high FPG and diabetes mellitus were risk factors for GSD^35–37^. GSD appeared strongly associated with fasting glycemia^38^. We noted that there was a positive correlation between prevalence of GSD and higher FPG, regardless of sex. The possible mechanisms for this association may be as follows: hyperglycaemia inhibits bile secretion from the liver and disturbs gallbladder contraction^39^; hyperglycaemia may affect gallbladder motility^40^; or some factors modifying the crystal nucleation and mucous secretion in bile^41^. In line with previous research reporting lower HDL-C to be a risk factor for GSD^17,18,33^, our study also arrived at this conclusion in the total sample. In agreement with our finding that lower HDL-C was associated with GSD in men but not in women, a study in Taiwanese population also found that low HDL-C was associated with GSD in men but did not found that FPG was related to GSD^18^.

It is well established that thyroid dysfunction, either hyperthyroidism or hypothyroidism was associated with higher prevalence of GSD^19,20^, however, in a euthyroid population, the association between TSH levels and GSD has never been investigated. In our study, we found in the population with normal thyroid function, the lower TSH level, the lower the prevalence of GSD (OR = 0.442, 95% CI 0.271–0.722). This phenomena was also found in women (OR = 0.329, 95% CI 0.145–0.744). However, in men, the difference was not significant, although the trend was obvious (OR = 0.601, 95% CI 0.247–1.460). These results indicated that even in participants with normal thyroid function, TSH still played an important role in gallstone formation.

The strengths of the study should be acknowledged. First, although sex differences in GSD or MetS were investigated in previous studies, the majority of those studies focused on sex differences in either GSD or MetS in the same study, and sex differences in the metabolic risk factors for GSD was rarely reported. To the best of our knowledge, our study was the first to comprehensively assess these sex differences. Second, all the participants included in the study underwent abdominal ultrasound examination, and the presence of gallstones and evidence of previous cholecystectomy were documented by imaging and not self-report, which enabled the diagnosis of GSD to be accurate, and further analysis was scientific and reasonable. Third, every participant in the current study was individual who had a routine physical check-up and had normal thyroid function, which may represent the majority of residents in mainland China, and for the participants who had one or more of above risk factors, it seemed to be possible to reduce the risk of GSD by lifestyle intervention.

Of course, there are several limitations in this study. First, it was a retrospective, cross-sectional study, so no causal relationships can be assessed. Second, certain recognized GSD risk factors, such as family history of gallstones, oral contraceptive use, number of pregnancies, and hepatitis C infection were not assessed in the present study. Third, some serum markers including serum insulin and leptin level, hepatic and peripheral insulin resistance, were not measured in the current study. Therefore, prospective studies with large sample sizes are warranted to determine the true relationship between MetS and GSD considering the above factors.

In conclusion, our analyses show that MetS is strongly associated with GSD in Chinese euthyroid population, and MetS components, including high FPG, low HDL-C, and low TSH levels are independent risk factors for GSD; however, there are possibly different risk factors for GSD in men and women. All these findings in our study should be further tested in a prospective, multi-centre study with a large sample size.

## Methods

### Study population

The study was approved by the Ethics Committee of Ningbo Medical Center Lihuili Hospital. All procedures performed in studies involving human participants were in accordance with the ethical standards of the institutional and/or national research committee and with the 1964 Declaration of Helsinki and its later amendments or comparable ethical standards. Due to the retrospective nature of this study and the decision of Ethics Committee of Ningbo Medical Center Lihuili Hospital, informed consent was not necessary for this study.

**Figure 2:**
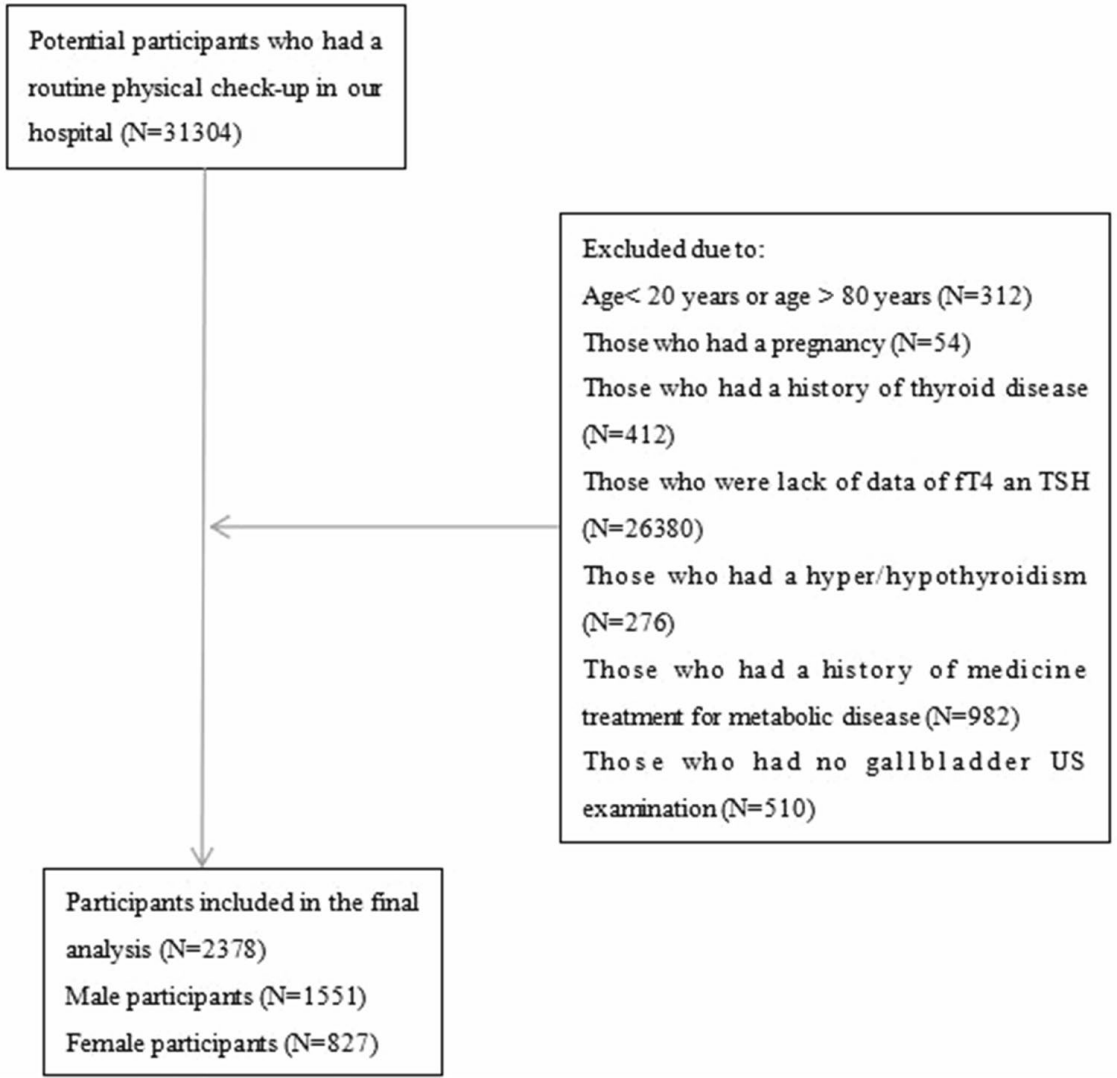
Flow chart of the study.

Doctor Du and Wang were the administrators of health examination centre and had necessary permissions to the database. Individuals who underwent a routine physical check-up between June 2016 and December 2017 were potential participants. The exclusion criteria were as follows: (1) age < 20 or > 80 years; (2) pregnancy or within the first year of the postpartum period; (3) subclinical/overt hyperthyroidism or subclinical/overt hypothyroidism; (4) history of thyroid disease, including history of surgery or medication); (5) history of one or more metabolic diseases such as hyperlipidaemia, hypertension, hyperglycaemia, hyperuricaemia and concurrent medication for these diseases. Figure 2 shows the flow chart of the included participants.

### Data collection

Considering that variables including sex, age and metabolic factors were potential risk factors for GSD, we retrospectively collected the following data from the health examination database: sex, age, height, weight, BMI, systolic blood pressure (SBP), diastolic blood pressure (DBP), fasting plasma glucose (FPG), total cholesterol (TC), high-density lipoprotein cholesterol (HDL-C), low-density lipoprotein cholesterol (LDL-C), triglycerides (TGs), serum creatinine (Scr), uric acid (UA), free triiodothyroxine (fT3), free tetraiodothyroxine (fT4) and TSH. Biochemical parameters including FPG, TG, HDL-C, LDL-C, UA, Scr, TSH, fT3 and fT4 were tested in fasting blood samples.

### Definition

Gallstones were diagnosed with ultrasonography if the gallbladder contained echoes that moved with gravity except when the stones were large, a septum existed in the gallbladder or there was an enclosed infundibulum^42^. GSD was defined as sonographically diagnosed gallstones or previous history of cholecystectomy. According to the criteria given by the Chinese Medical Association Diabetes Branch (CDS) designed for Chinese^43^, MetS was defined as presence of three or more of the following four risk factors: (1) obesity/overweight, BMI ≥ 25.0 kg/m^2^; (2) hypertension, SBP ≥ 140 mmHg or/and DBP ≥ 90 mmHg or previous diagnosis; (3) dyslipidaemia, defined as fasting TG ≥ 1.7 mmol/L or HDL-C < 0.9 mmol/L in men, HDL-C < 1.0 mmol/L in women; (4) hyperglycaemia, defined as FPG ≥ 6.1 mmol/L. Euthyroid was defined as a normal serum fT4 level with a normal serum TSH level. In our hospital, TSH was measured using an E-TSH kit (Roche Diagnostics), for which the reference range was 0.50–5.00 μIU/mL.

### Statistical analysis

All the participants were categorized into two groups, the GSD group and non-GSD group, based on the presence of GSD. In this study, the following analyses were performed in males, females and the general population. Between-group comparisons were performed using the χ^2^ (chi-square) test for categorical variables and a t test for continuous variables (mean ± standard deviation, M ± SD). Then, multivariate logistic regression analysis adjusting for variables that were associated with GSD in the univariate analysis was performed to test factors’ independence. Statistical analyses were performed using SPSS 19.0 software (SPSS, Chicago, IL, http://www.spss.com). The statistical test were two-sided. P value less than 0.05 was considered statistically significant, and the odds ratio (OR) and 95% confidence interval (CI) were also calculated.

## Data Availability

All the data used and analyzed are available from corresponding authors upon the reasonable request.
